# Validation of the IBIS breast cancer risk evaluator for women with lobular carcinoma in-situ

**DOI:** 10.1038/s41416-018-0120-z

**Published:** 2018-06-21

**Authors:** Louisa Lisa Lo, Roger Laughlin Milne, Yuyan Liao, Jack Cuzick, Mary Beth Terry, Kelly-Anne Phillips

**Affiliations:** 1 Division of Cancer Medicine, Peter MacCallum Cancer Centre, Melbourne, VIC Australia; 2 Cancer Epidemiology and Intelligence Division, Cancer Council Victoria, Melbourne, VIC Australia; 3 Centre for Epidemiology and Biostatistics, Melbourne School of Population and Global Health, The University of Melbourne, Melbourne, VIC Australia; 4 Department of Epidemiology, Columbia University Mailman School of Public Health, New York, NY 10032 USA; 5 Centre for Cancer Prevention, Wolfson Institute of Preventive Medicine, Queen Mary University of London, London, UK; 6 Herbert Irving Comprehensive Cancer Center, Columbia University, New York, NY 10032 USA; 7 Sir Peter MacCallum Dept of Oncology, The University of Melbourne, Parkville, VIC, 3053 Australia; 8 Department of Medicine, St Vincent’s Hospital, The University of Melbourne, Parkville, VIC, 3053 Australia

**Keywords:** Breast cancer, Cancer prevention

## Abstract

**Background:**

Management advice for women with lobular carcinoma in situ (LCIS) is hampered by the lack of accurate personalised risk estimates for subsequent invasive breast cancer (BC). Prospective validation of the only tool that estimates individual BC risk for a woman with LCIS, the International Breast Cancer Intervention Study Risk Evaluation Tool (IBIS-RET), is lacking.

**Methods:**

Using population-based cancer registry data for 732 women with LCIS, the calibration and discrimination accuracy of IBIS-RET Version 7.2 were assessed.

**Results:**

The mean observed 10-year risk of invasive BC was 14.1% (95% CI:11.3%-17.5%). IBIS-RET overestimated invasive BC risk (p = 0.0003) and demonstrated poor discriminatory accuracy (AUC 0.54, 95% CI: 0.48 – 0.62).

**Conclusions:**

Clinicians should understand that IBIS-RET Version 7.2 may overestimate 10-year invasive BC risk for Australian women with LCIS. The newer IBIS-RET Version 8.0, released September 2017, includes mammographic density and may perform better, but validation is needed.

## Introduction

Women diagnosed with lobular carcinoma in-situ (LCIS) have an elevated risk of subsequent invasive breast cancer (BC)^1^ that increases by about 1% every year after diagnosis to 13% after 10 years, 11%-26% at 15 years^1–3^ and 21%-26% risk after 20 years.^4, 5^

Most are managed with observation alone,^6^ but American Society of Clinical Oncology and Cancer Australia guidelines recommend that risk-reducing medications, specifically selective oestrogen receptor modulators (SERM) or aromatase inhibitors (AI) (the latter only in postmenopausal women), be discussed with LCIS patients.^7–10^ Risk-reducing bilateral mastectomy is pursued by only a minority of LCIS patients.^11^ Informed decision-making would be facilitated by accurate personalised risk estimates for future invasive BC.

The International Breast Cancer Intervention Study Risk Evaluation Tool (IBIS-RET) is the only tool available to estimate risk for an individual woman with LCIS. ^12–14^ Although validated in other populations,^15–17^ IBIS-RET, to our knowledge, has not been validated in LCIS patients. Using population-based data, we prospectively examined the performance of IBIS-RET Version 7.2 for estimating invasive BC risk for women with a history of LCIS.

## Methods

The Victorian Cancer Registry (VCR) has collected data on all cancer diagnoses in Victoria, Australia since 1982. It determines vital status of all registrants by record linkage to the state and national death registries.^18^ De-identified data including dates of birth, death, LCIS and invasive BC diagnoses were obtained from the VCR for all women diagnosed with pure LCIS between 1982 and 2015, when aged 20–70 years. ‘Pure’ LCIS was defined as LCIS without previous or synchronous ductal carcinoma in-situ (DCIS) or invasive BC in either breast (including within 6 months after LCIS diagnosis). Women with other invasive cancer diagnoses (except non-melanotic skin cancer) prior to their pure LCIS diagnosis were excluded. The study was approved by the Peter MacCallum Cancer Centre ethics committee.

The calibration and discriminatory accuracy of the 10-year IBIS-RET Version 7.2 estimates were assessed by comparing IBISRET-assigned risks with observed invasive BC incidence. To assess calibration, the mean IBIS-RET-assigned risk was compared with the mean 10-year observed invasive BC incidence in each IBIS-RET-assigned risk group, using a chi-squared goodness-of-fit statistic,^19^ for the whole cohort (by tertiles) and also for two subgroups stratified by the diagnosis of LCIS before and at or after age 50 years. To evaluate discriminatory accuracy, the overall area under the receiver operating characteristic (ROC) curve for the development of invasive BC within 10 years of LCIS diagnosis was computed. RMAP (https://gailg.github.io/rmap/) and SAS software 9.4 (SAS Institute, Cary, NC) were used. Data were censored at date of invasive BC diagnosis, death and date that the most recently linked death data were considered complete (31^st^ December 2015). Two exploratory analyses were also conducted; the first censored the data at date of any ductal carcinoma in situ (DCIS) diagnosis and the second included diagnosis of either invasive breast cancer and/or DCIS as the primary endpoint.

## Results

There were 732 eligible women (median age at LCIS 50 years, range 25–70 years, mean follow-up 9.8 years, range 0.04–33.9 years, total 4855 person-years), of whom 73 were diagnosed with invasive BC within 10 years after their LCIS. 10 women died within 10 years without an invasive BC diagnosis, 293 women were invasive BC-free at 10 years and 356 women were last observed without invasive BC with less than 10 years follow-up. The mean observed risk of invasive BC at 10 years was 14.1% (95% confidence interval (CI) 11.3%-17.5%), whilst the mean assigned IBIS-RET 10-year risk was 20.9%.

**Fig. 1:**
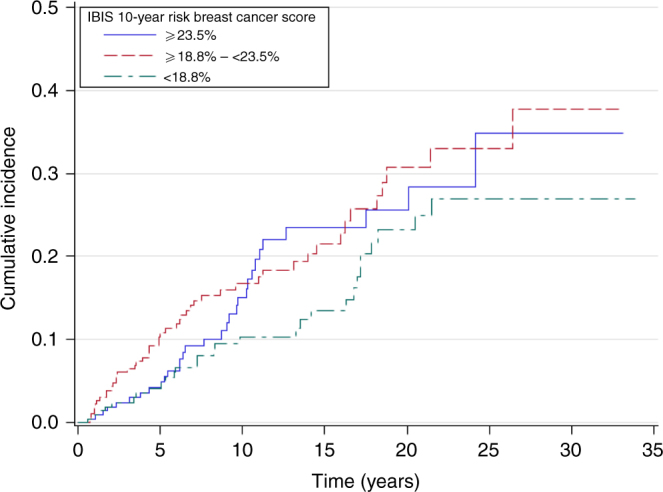
Cumulative incidence of invasive breast cancer in patients with LCIS, by tertile of IBIS-RET estimated risk

Figure 1 shows the cumulative invasive BC incidence by 10-year IBIS-RET-assigned risk tertile (i.e. <18.8%, ≥18.8%–<23.5%, ≥23.5%).

**Fig. 2:**
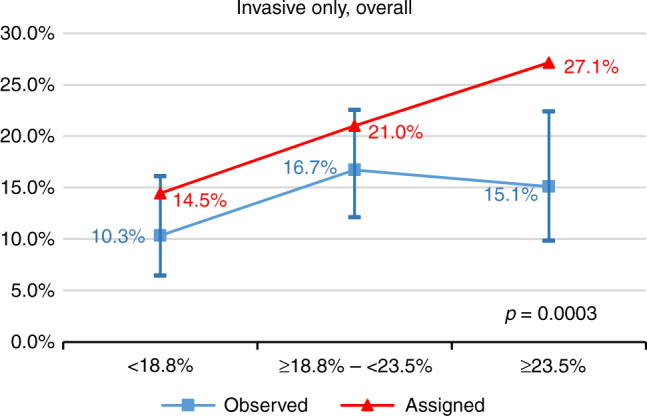
Calibration of IBIS-RET for estimates of invasive BC in women with LCIS. The assigned line (triangle symbol) is the mean 10-year predicted risks of the IBIS-RET for that tertile (<18.8%, ≥18.8%–<23.5%, ≥23.5%). The observed line (square symbol) is the estimates of 10-year breast cancer probabilities based on the womens' observed breast cancer status, and the bars denote 95% confidence intervals for the observed risk

**Fig. 3:**
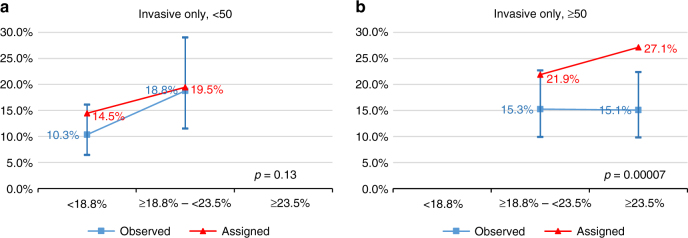
**a** Calibration of IBIS-RET for estimates of invasive BC in women with LCIS diagnosed at <50 years (cut-off: <18.8%, ≥18.8%–<23.5%). **b** Calibration of IBIS-RET for estimates of invasive BC in women with LCIS diagnosed at >=50 years (cut-off: ≥18.8%–<23.5%, ≥23.5%). The assigned line (triangle symbol) is the mean 10-year predicted risks of the IBIS-RET for that tertile (<18.8%, ≥18.8%-<23.5%, ≥23.5%). The observed line (square symbol) is the estimates of 10-year breast cancer probabilities based on the womens’ observed breast cancer status, and the bars denote 95% confidence intervals for the observed risk

Figure 2 shows that the mean IBIS-RET-assigned invasive BC risks by tertile were significantly different to the observed BC incidence at 10 years (p = 0.0003). Overall the IBIS-RET Version 7.2 tended to overestimate invasive BC risk. When we compared the calibration for women below and above age 50 using internal cutpoints by age, we found it was well-calibrated for women diagnosed before age 50 years (p = 0.13), but not for those diagnosed at or after age 50 years mainly owing to the lack of fit and overestimation by IBIS-RET for older women in the highest quantile, (p = 0.00007) (Fig. 3a,b).

The area under the ROC curve (AUC) for the IBIS-RET 10-year invasive BC risk estimates was 0.54 (95% CI: 0.48 – 0.62) overall, and 0.55 (95% CI: 0.44 – 0. 0.63) and 0.48 (95% CI: 0.38 – 0.57) for women diagnosed with LCIS before age 50 years and at or after age 50 years, respectively.

Nine women were diagnosed with DCIS during the first 10 years after their LCIS diagnosis. The results were similar when the analyses were repeated either censoring at DCIS diagnosis or including the 9 cases of DCIS along with the invasive BC cases in the primary endpoint (Supplementary Figure 1).

## Discussion

In this population-based study of women with LCIS, the IBIS-RET Version 7.2 tended to overestimate 10-year invasive BC risk and had poor discriminatory accuracy. However this study had several limitations that may have contributed to this finding.

The lack of information in our dataset regarding uptake of bilateral mastectomy or risk-reducing medication after LCIS diagnosis could have resulted in our study erroneously finding that IBIS-RET overestimates BC risk. However, uptake of these interventions is historically very low in Australia, even in very high-risk women,^20^ so this is unlikely to have been a major factor.

Histopathological diagnostic thresholds for atypical hyperplasia (AH) and LCIS have changed over time.^21^ If some of the cases included in this study were in fact AH misclassified as LCIS, this could have contributed to our finding that IBIS-RET V7.2 overestimates BC risk, because AH confers a lower BC risk than LCIS. No pathology review of cases was undertaken by the authors or the VCR.

The IBIS-RET model is calibrated to UK BC incidence rates for 2008–2010 (Supplementary Table 1).^22^ Although our study covers Victorian women over a period from 1982–2015, the average age-specific BC incidence figures for these Victorian women closely resembled those used in the IBIS-RET model, except for the lower incidence in those aged 50 years and over (Supplementary Table 1).^23^ This could have contributed to the overestimation and poorer calibration in our dataset for women diagnosed with LCIS at and over age 50 years, but it is unlikely to completely explain our findings.

Overdiagnoses from mammographic screening could have influenced our findings. However, Victorian women aged 50 years and over are screened 2-yearly as opposed to 3-yearly in the UK and this higher screening frequency should, if anything have resulted in a higher invasive BC incidence when compared to the UK population, but this was not observed in those aged older than 50 years (Supplementary Table 1).

Patient migration out of Victoria after LCIS diagnosis would mean that some subsequent invasive breast cancers were not captured in the VCR data. Using aggregate data from the Australian Institute of Health and Welfare, we estimated that this interstate migration would have resulted in approximately 3 cases of invasive cancer being missed in our dataset, which could in part have contributed to our finding that IBIS-RET overestimates BC risk.

The study dataset also did not include information on other BC risk factors, but because IBIS-RET Version 7.2 relies only on age to estimate subsequent BC risk in women with LCIS, this would not have impacted our findings. In fact there are conflicting reports on whether age at LCIS diagnosis affects subsequent BC risk. One study reported that the relative risk of BC tended to decrease with increasing age at LCIS diagnosis.^4^ Conversely, according to King et al,^3^ risk factors like family history, age and breast density were not associated with BC risk in women with LCIS. Instead, the authors found that chemoprevention was the major factor associated with lower BC risk (Hazard ratio, 0.27; 95% CI, 0.15 to 0.50). They also performed a subgroup nested case-control analysis, which showed that the volume of LCIS, which was defined as the ratio of slides with LCIS to total number of slides reviewed, was associated with BC development (p = 0.008). Therefore, volume of LCIS might provide further risk stratification in women with LCIS.

Mammographic density is an important risk factor for breast cancer and has been shown to refine the IBIS-RET model in predicting BC risk for women at increased risk, although not specifically in women with LCIS.^24^ A subsequent study using a UK prospective BC screening cohort showed that using mammographic density with the IBIS-RET improved the accuracy of BC risk prediction.^25^

Since starting this study, IBIS-RET, Version 8 ^22^ has been released (September 2017). For women with LCIS, Version 8 differs from Version 7.2 in that it now uses cancer family history and mammographic density (if available) to predict BC risk, as well as age at diagnosis of LCIS. The addition of cancer family history means that, in the absence of mammographic density information, IBIS-RET Version 8 will always provide the same or higher 10-year BC risk estimates as Version 7.2 (which we have shown here already tends to overestimate risk). However, if mammographic density is known to be low, the risk estimate provided by Version 8 may be lower than that provided by Version 7.2.^25^ A validation study of IBIS-RET Version 8, using a dataset of women with LCIS and known mammographic density and family history, is highly desirable.

## Electronic supplementary material

Supplementary Figure 1. Calibration of IBIS-RET for estimates of BC (invasive or DCIS) in women with LCIS

Supplementary Table 1. Average age-specific breast cancer incidence (per 100,000 women) for the Victorian population from 1982 to 2015 compared to those used in IBISRET model v7/8

## Availability of data and material

The data used for this study are available from the Victorian Cancer Registry but restrictions apply to the availability of these data, which were used for the current study with appropriate approvals, and so are not generally publicly available. Data are however available from the authors upon reasonable request and with permission of the Victorian Cancer Registry.
