# Supplementary material for: Validation of the IBIS breast cancer risk evaluator for women with lobular carcinoma in-situ
Source: Br J Cancer. 2018 Jun 21;119(1):36–9. doi: 10.1038/s41416-018-0120-z (PMC6035272; doi:10.1038/s41416-018-0120-z)
Supplement: Supplementary file 1 — Supplementary Figure 1. Calibration of IBIS-RET for estimates of BC (invasive or DCIS) in women with LCIS [file 41416_2018_120_MOESM1_ESM.docx]

**Supplementary Figure 1. Calibration of IBIS-RET for estimates of BC (invasive or DCIS) in women with LCIS.**

| **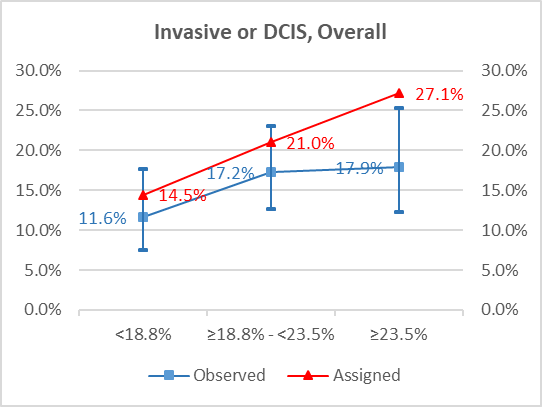** |
| --- |
| 1A. Risk Plot Goodness of Fit for invasive BC or DCIS cases, between observed and assigned IBIS risk, overall, 3.571, *p*-value= 0.01. |
| **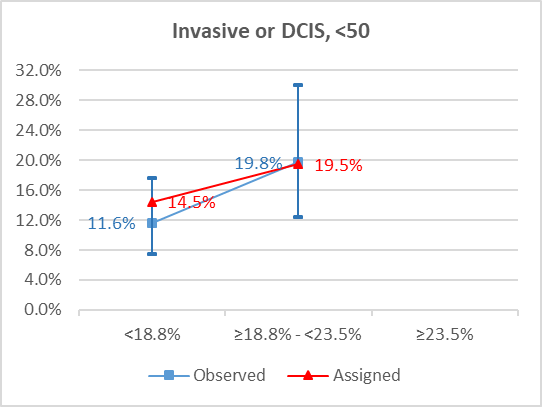** |
| 1B. Risk Plot Goodness of Fit for invasive BC or DCIS cases, between observed and assigned IBIS risk, in those < 50 years, 0.846, *p*-value= 0.41. |
| **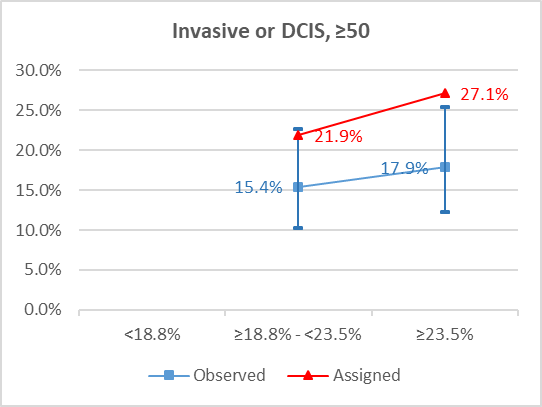** |
| 1C. Risk Plot Goodness of Fit for invasive or DCIS cases, between observed and assigned IBIS risk, in those ≥ 50 years, 6.2, *p*-value= 0.002. |
