# Supplementary material for: Validation of the IBIS breast cancer risk evaluator for women with lobular carcinoma in-situ
Source: Br J Cancer. 2018 Jun 21;119(1):36–9. doi: 10.1038/s41416-018-0120-z (PMC6035272; doi:10.1038/s41416-018-0120-z)
Supplement: Supplementary file 2 — Supplementary Table 1. Average age-specific breast cancer incidence (per 100,000 women) for the Victorian population from 1982 to 2015 compared to those used in IBISRET model v7/8 [file 41416_2018_120_MOESM2_ESM.doc]

Supplementary Table 1. Average age-specific breast cancer incidence (per 100,000 women) for the Victorian population from 1982 to 2015 compared to those used in IBISRET model v7/8 ^22, 23^

| **Age Group** | **IBISRET v7/8 (UK)** | **Victoria (1982 to 2015)** |
| --- | --- | --- |

| 20 – 24 | 1.3 | 1.2 |
| --- | --- | --- |
| 25 – 29 | 9.1 | 8.4 |
| 30 – 34 | 24.2 | 26.3 |
| 35 – 39 | 57.5 | 59.5 |
| 40 – 44 | 115.6 | 118.0 |
| 45 – 49 | 182.3 | 179.3 |
| 50 – 54 | 250.3 | 224.0 |
| 55 – 59 | 266.2 | 249.0 |
| 60 – 64 | 318.7 | 286.2 |
| 65 – 69 | 378.5 | 315.9 |
| 70 – 74 | 296.4 | 304.6 |
| 75 – 79 | 331.9 | 302.2 |
| 80 – 84 | 369.7 | 317.7 |
